# A Diagnostic Challenge: Varicella-Zoster Virus Meningoencephalitis in a Young Medical Student

**DOI:** 10.7759/cureus.84237

**Published:** 2025-05-16

**Authors:** Michael Zatoulovski, Jacob de Castro, Zulfiya Tashpulatova, Gabor Legradi

**Affiliations:** 1 Internal Medicine, William Carey University College of Osteopathic Medicine, Hattiesburg, USA; 2 Psychiatry, William Carey University College of Osteopathic Medicine, Hattiesburg, USA; 3 Neurology/Behavior Science, William Carey University College of Osteopathic Medicine, Hattiesburg, USA

**Keywords:** autobiographical case report, herpes virus encephalitis, somatic symptoms and related disorders, varicella meningitis, vestibular neuritis

## Abstract

Varicella-zoster virus (VZV) meningoencephalitis is an incredibly rare complication following human herpesvirus-3 (HHV-3) infection, primarily diagnosed in children and those who are immunocompromised. The gold standard for diagnosing this illness is polymerase chain reaction (PCR) testing of the cerebrospinal fluid (CSF) after obtaining it through a lumbar puncture, and treating the infection promptly with acyclovir and glucocorticoids.

In this case report, we present a 24-year-old male medical student with nonspecific neurological symptoms. The patient's initial presentation included headaches, fatigue, and cognitive difficulties. Over a three-month period, the patient underwent multiple MRI and CT scans with the assumption that the underlying etiology was autoimmune or chronic in nature, and not something that would be caused by a pathogen of any kind. Persistence of his symptoms eventually prompted a lumbar puncture, revealing VZV in the cerebrospinal fluid and confirming the diagnosis of meningoencephalitis.

This case emphasizes how VZV should remain on the list of differential diagnoses no matter how long the symptoms have persisted because the virus's nature is to remain latent and reactivate in situations of extreme stress. Therefore, if suspected, a lumbar puncture should not be delayed in order to diagnose and treat the condition promptly. Finally, because most cases of this kind of meningoencephalitis are treated within a week of onset of symptoms, we present this case to explore the long-term side effects of VZV encephalitis and its disease progression if it goes untreated.

## Introduction

Varicella-zoster virus (VZV), an alpha-herpesvirus commonly known for causing chickenpox, can lead to acute complications, such as encephalitis, sepsis, pneumonia, and even cerebral hemorrhage [1]. Typically, VZV establishes latency in neurons after a primary infection and is later reactivated due to stress, immunosuppression, or other factors [2-3]. Common symptoms of VZV encephalitis include fever, headache, seizures, and altered mental status, often accompanied by a rash and skin vesicles. Neuropsychiatric features such as behavioral changes, hallucinations, and cognitive decline can also occur [4]. If suspected, neuroimaging and lumbar puncture (LP) are crucial early diagnostic tools in assessing patients with viral encephalitis. CT or MRI scans are used to rule out elevated intracranial pressure before conducting an LP [4]. This case report delves into the unique presentation of VZV encephalitis in a 24-year-old likely due to heightened stress levels. This case is also important because of the lack of dermatological symptoms and low suspicion of the differential diagnosis. 

## Case presentation

History of presentation

 A 24-year-old male patient presented to the emergency room complaining of a sharp, pounding headache on the left side with associated photophobia, tinnitus, and vertigo that started earlier that day. He says that nothing could make the headache better or worse and that it just feels like "the worst headache he has ever felt in his life." He stated that two weeks ago he was diagnosed with a middle ear infection and was treated with a course of azithromycin. However, after a week, the infection did not go away, and he was prescribed a subsequent course of cefdinir, which, after a second week, also failed to alleviate his symptoms. He described his symptoms as feeling fluid in his ear that he could not get out, and that it caused an itch sensation closer and closer to his inner ear. His other past medical history was unremarkable except for a bout of myocarditis secondary to coxsackie B virus at age 16. His surgical history is relevant for the removal of tonsils in childhood, and the removal of a umbilical hernia at age 7. He does not smoke, consume alcohol, or take recreational drugs, and is sexually active in a long-term monogamous relationship with consistent condom use. He provided transcripts confirming that he was up to date on all of his childhood, flu, and COVID-19 vaccines. Aside from his chief complaint, his review of symptoms was positive for abdominal pain and severe, constant diarrhea for the past week, for seemingly no attributable reason. He has not traveled outside of the country recently, and is a full-time medical student. 

On physical exam, the patient was alert and oriented to person, place, and time. His cranial nerves II-XII were intact, and his left ear showed air fluid levels beyond the tympanic membrane. His gait was unremarkable. A Romberg test was negative with the patient's eyes open and positive when his eyes were closed. The patient was able to perform a finger-to-nose touch, but had difficulty doing a heel-to-shin test due to some balance instability. Sensory, motor, and proprioception sensations were intact throughout. He had a negative Kernig and Brudzinski test. All reflexes were 2+, and a detailed exam of all other physical systems was unremarkable.

Investigations and results

ED Admission

**Figure 1:**
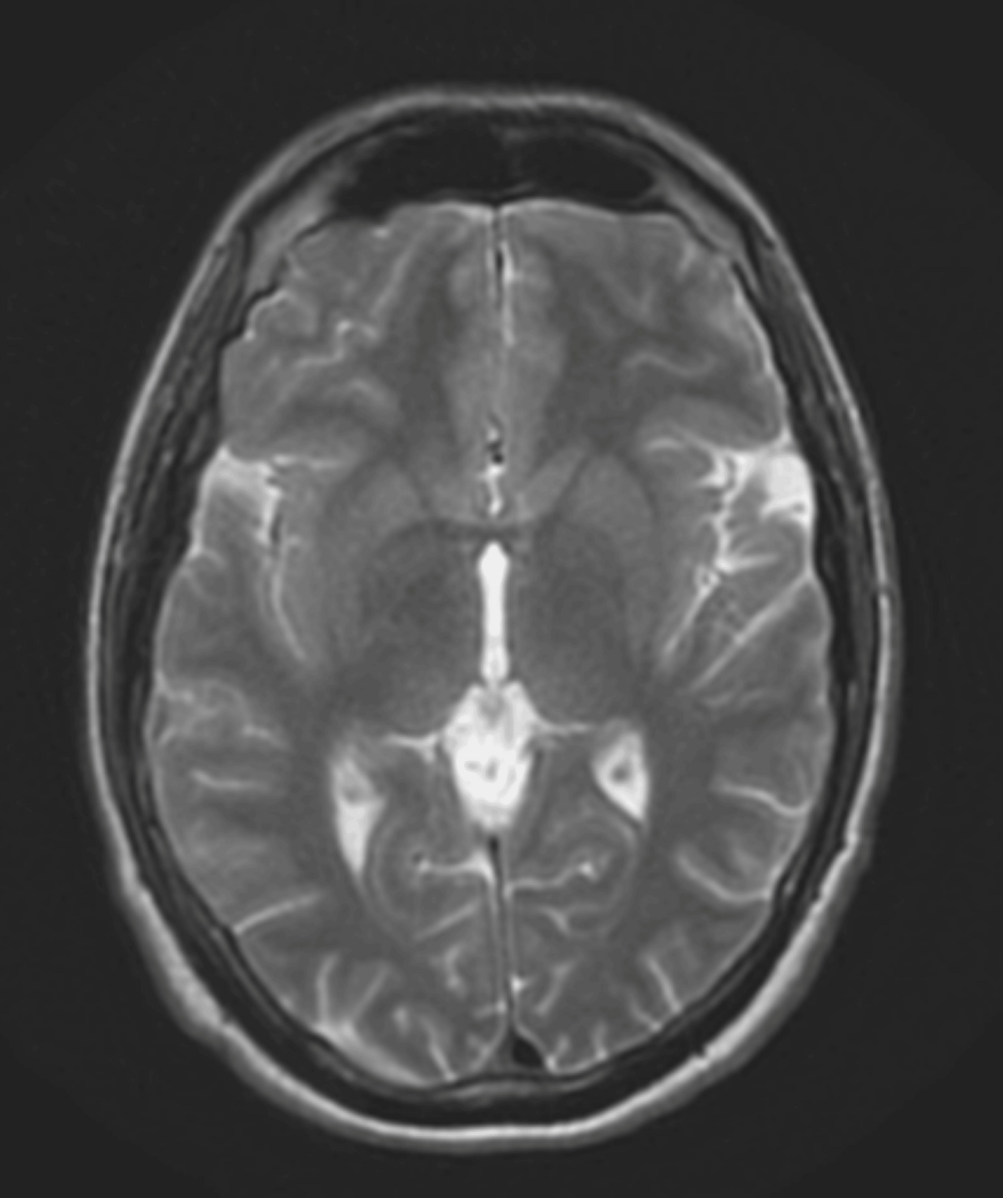
T2-weighted MRI of the brain with and without contrast (11/14/22) Impression: No significant intracranial process. No acute abnormality. Clinical examination: 24-year-old male with meningitis and elevated intracranial pressure

In the emergency department, a complete blood count and comprehensive metabolic panel were drawn, which showed an elevated white count of 14 and a potassium level of 2.9. A non-contrast CT and an MRI without contrast were performed as well during the same hospital stay and were read to be unremarkable (Figure 1). The patient was given IV potassium supplementation while staying in the hospital overnight. He was discharged with a diagnosis of vestibular neuritis and migraines secondary to prolonged otitis media with a course of levofloxacin 500mg and was referred to an ENT for follow-up as an outpatient.

ENT Follow Up (One Day After ER Discharge)

During the ENT follow-up appointment, the patient felt incredibly fatigued. He stated that his symptoms persisted except for his diarrhea, which resolved, and his headache, which slightly improved after discharge. At the ENT, a thorough examination of his left ear showed no erythema or signs of an ear infection of any kind. Furthermore, an audiogram was performed, and demonstrated mild loss of higher frequency hearing. Concerned about his persistent symptoms, the ENT referred him to another ENT at an academic hospital center for a second opinion.

ENT Follow Up 2 (Three Weeks After ER Discharge)

At the academic ENT outpatient office, the patient confided that he was starting to feel like this illness had altered his personality. He stated that he had never felt such anxiety or depression before in his entire life. Furthermore, he explained that he has started to develop paranoia and jealousy, which he emphasized had never been a part of his personality before. In terms of his physical symptoms, he stated that they continued to persist and that, in addition to the fatigue, he developed a dull, stabbing pain in the back of his head around the area of the occiput and upper neck. He stated that he felt stiff in that area, and at times it felt like "someone literally hit him over the head with a frying pan." Concerned over the unrelenting vertigo symptoms, the ENT decided to get a brain MRI, this time with contrast, and performed an intratympanic steroid injection in the hopes of relieving what she thought to be residual inflammation within the inner ear. The patient was then told to follow up via telemedicine in one week regarding symptoms and was referred to a psychiatrist.

Psychiatrist Follow Up (Six Weeks After ER Discharge)

Throughout the psychiatry appointment, the patient showed clear signs of anxiety and depression. He had difficulty concentrating, trouble sleeping, lost interest in his usual hobbies, and was constantly worried. He also felt guilty about taking a leave of absence from medical school, even though he had not been diagnosed with any condition. Without any clear objective findings behind the patient's symptoms, the patient was diagnosed with brief illness anxiety disorder and was prescribed fluoxetine 10mg, mirtazapine 7.5mg to help with sleep, and was told to follow up with his primary care provider (PCP) for his symptoms.

PCP Follow Up (Eight Weeks After ER Discharge)

The patient still appeared very mentally distressed, and was reassured to continue taking the fluoxetine as it would take a month or two before it could start working. Regarding his physical symptoms, the patient said that the vertigo was slowly resolving, but that it was still difficult for him to focus his vision like before. He described his vision difficulties as "not being able to look at the whole room, but only one object at a time." In addition, the patient was still experiencing extreme fatigue, dull global headaches daily, pain in the back of his head and neck, and tinnitus. The PCP took a basic CBC, CMP, and thyroid panel, and referred the patient to an ophthalmologist and rheumatologist to rule out any autoimmune causes for the patient's symptoms.

Rheumatology Follow Up (10 Weeks After ER Discharge)

Recognizing the patient's emotional and physical turmoil, the rheumatologist ordered a battery of tests to begin ruling out anything she thought could cause such symptoms. This included a Lyme disease panel, anti-nuclear antibody, erythrocyte sedimentation rate, C-reactive protein, herpes simplex antigen and PCR, varicella zoster IgG and IgM, SARS-CoV-2 IgG and IgM, heterophile antibody, thiamine, folic acid, Vitamin B12, and a complement panel. All of which returned within normal limits. When taking a more thorough history, the patient explained how a few weeks before his symptoms, he was traveling and visited some crowded amusement parks with several potential sick contacts, where he experienced some upper respiratory symptoms. However, he had assumed this was due to an ongoing middle ear infection and did not think anything more of it at the time. The rheumatologist considered a diagnosis of long COVID and prescribed a methylprednisone-dose pack in an effort to tackle any residual inflammation left in the patient's body. He was then told to follow up with his PCP.

Ophthalmologist Follow Up (11 Weeks After ER Discharge)

At the ophthalmologist's office, the patient first had his vision tested. The patient stated that he last had his vision tested approximately nine months prior at his PCP's office and that he had always had 20/20 vision in both eyes. However, after evaluating his vision, it was determined that the patient had 20/20 vision in his left eye and 20/30 vision in his right eye. The tonometer read an intraocular pressure of 15 in the left eye and 22 in the right eye, and an examination of the eyes when dilated revealed some congenital drusen bilaterally, and mild cupping of the right optic disc. The ophthalmologist considered this to be a reason for the patient's visual problems, as the left eye would be working harder to compensate for the right eye. However, because the patient was still experiencing photophobia, he was referred to a neuroophthalmologist for further evaluation and was offered a pair of glasses to correct his vision.

Neuroophthalmologist Follow Up (15 Weeks After ER Discharge)

In the office, the patient stated that his symptoms continued to persist, but that they did slightly improve with the methylprednisolone. So much so that he requested a refill because he said even though he was tapering himself off it per the instructions, he noticed that as soon as he stopped, the symptoms would return with full force. After a thorough review of the patient's recent medical history and another eye examination showing slight papilledema of the right eye, the neuroophthalmologist decided to order a lumbar puncture with the suspicion of idiopathic intracranial hypertension. Along with checking the opening pressure, standard meningitis, neurosyphilis, and oligoclonal bands were labs ordered (Table 1). The lumbar puncture was performed via fluoroscopy, and initially the opening pressure was only 4cm H2O, but a second attempt showed an opening pressure of 32cm H2O. Thinking that this may be the cause of all the symptoms, the patient was prescribed acetazolamide 125mg tablets and was told to follow up in two weeks to see how he was feeling. However, a few days later, the patient received a phone call telling him to urgently return to the hospital because the meningitis panel returned and was positive for VZV.

**Table 1:** CBC with differential (3/28/2023) performed after lumbar puncture showed evidence of varicella zoster meningitis. Results did not show any evidence of acute infection.

Test	Result	Reference value	Units
Granulocytes (absolute)	4.1	1.4-6.5	K/μL (x10^3/μL)
Lymphocytes (absolute)	2.5	1.2-3.4	K/μL (x10^3/μL)
Monocyte (absolute)	0.70	0.11-0.59	K/μL (x10^3/μL)
Eosinophils (absolute)	0.10	0.00-0.70	K/μL (x10^3/μL)
Basophils (absolute)	0.10	0.00-0.20	K/μL (x10^3/μL)
Absolute Neutrophil count	4,100	1,400-6,500	μL

Second Hospital Admission (16 Weeks After First ER Discharge)

**Figure 2:**
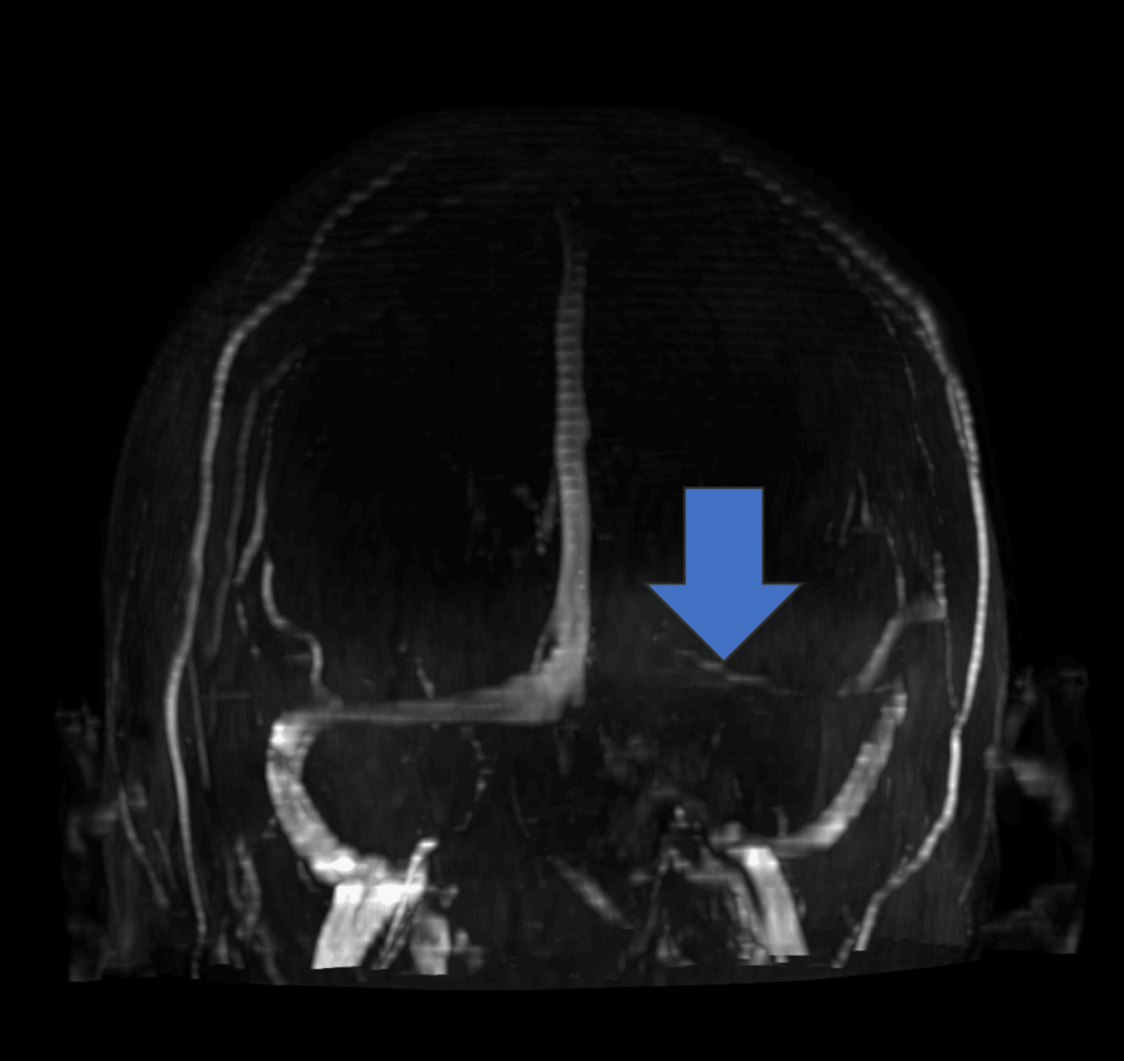
MRV head with and without contrast (3/29/23) Impression: (1) The arrow represents an absence of flow and enhancement of the right straight sinus. This is favored to be due to a developmental variant rather than thrombus, as this vessel is not well seen on brain MRIs. (2) No other acute conclusions of the venous structures.

**Figure 3:**
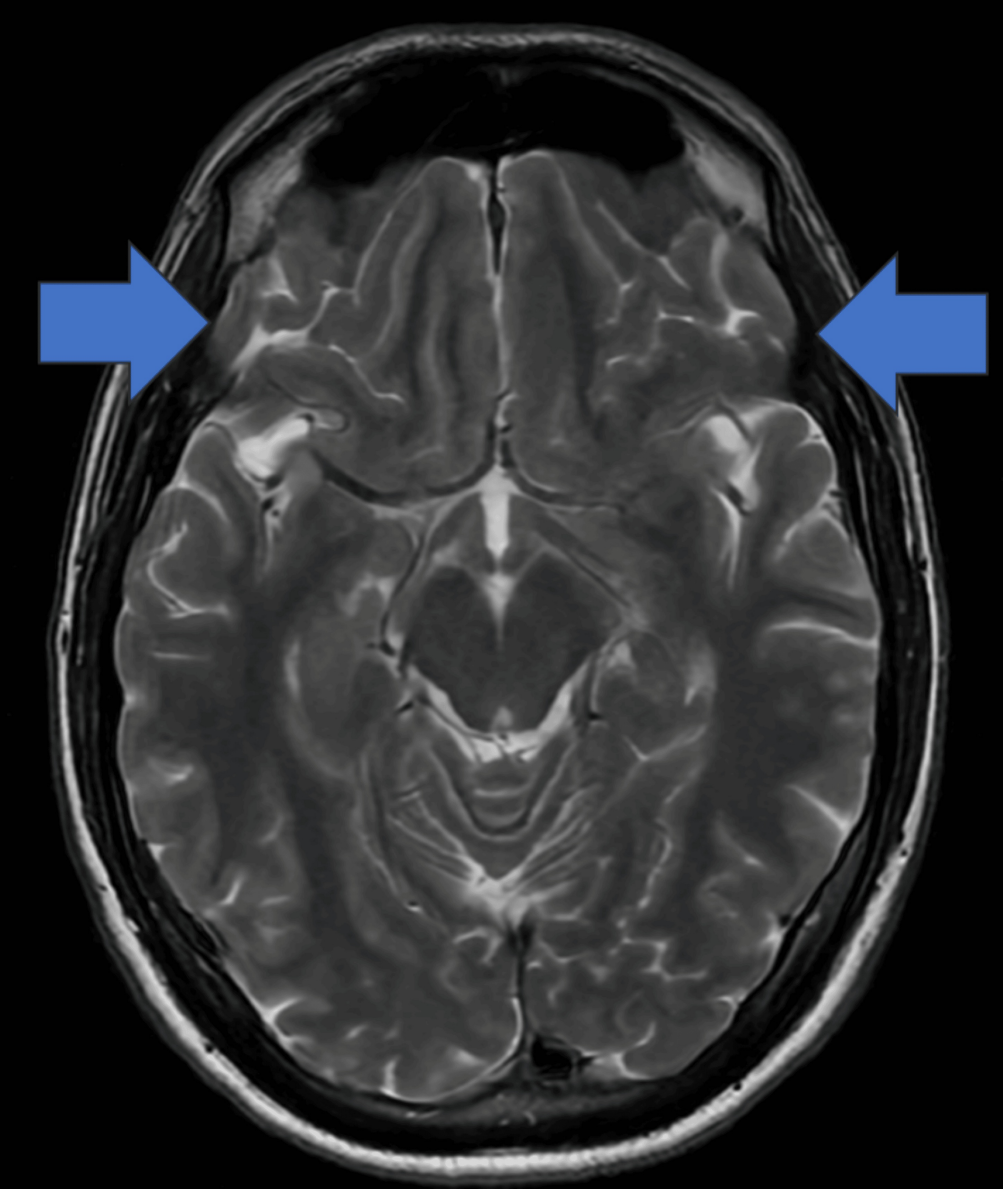
T2-weighted MRI of the brain with and without contrast (3/29/23) Impression: (1) The arrows represent the minimal increased dural and leptomeningeal enhancement in the bilateral frontotemporal regions. (2) No MR evidence for increased intracranial pressure was shown.

In the hospital ER, the patient was immediately given IV acyclovir. A second PCR was sent to confirm the initial diagnosis and was found once again to be positive for VZV. All other CSF panels were negative, and a pathology report showed no organisms with only a few nucleated cells present. On admission, a repeated CBC and CMP panel was performed and compared to his first admission (Table 2). Lastly, to observe the extent of the inflammation, a follow-up MRI, MRA, and MRV with and without contrast were performed, which confirmed the diagnoses as well as a coincidental hereditary finding (Figures 2-3).

**Table 2:** Lumbar puncture analysis (3/28/23) showing positive varicella-zoster virus antigens in the CSF

Test	Result	Reference Value
Haemophillus influenza	Negative	Negative
Neiserria meningitidis	Negative	Negative
Enterovirus	Negative	Negative
Herpes simplex virus 1	Negative	Negative
Varicella zoster virus	Detected	Negative

Management

With the MRI confirming inflammation of the leptomeninges, the patient was put on a four-week regimen of intravenous acyclovir 600mg and was discharged with a midline to continue treatment at home. After finishing the medication course, the patient was instructed to continue to take 1gm of oral valacyclovir daily for prophylaxis to prevent the virus from reactivating. Because the virus was active for so long, a repeat lumbar puncture was set to be performed three months after finishing the intravenous medication. When it was performed, the opening pressure was shown to be only 12cm H2O, and all concurrent panels, including the meningitis panel, were shown to be negative. On follow up with the patient's PCP the patient stated that his symptoms have markedly improved, and although he still feels residual fatigue, tinnitus, and headaches after long exhaustive days he states that his sleep has improved greatly, his ability to concentrate has returned, and most importantly he stated that "he feels like his old self again, and that the immense improvement happened so quickly after starting his intravenous treatment."

## Discussion

Most cases of VZV commonly present as reactivated shingles, usually in those aged 65 and above. While uncommon, the virus has been well-documented to progress to cause meningitis and encephalitis, producing classic symptoms of fever, headache, and altered mental status. Generally, this has been documented as secondary to immunosuppression [5]. In the few documented cases where VZV encephalitis occurred in children and young adults, the documented patients were immunocompromised patients, and were given IV acyclovir immediately, and subsequently had a very positive and full recovery [6]. Viral encephalitis is known to have a longer but less serious course than its bacterial counterpart [7]. 

In this case, we have a rare case of a patient suffering from VZV meningoencephalitis, and because the patient was not immediately treated with acyclovir, we were able to observe the indolent course of untreated VZV meningoencephalitis. Initially, when the patient first presented with symptoms, his MRI with and without contrast showed minimal to no evidence of acute encephalitis or inflammation in the brain. In addition, there was an absence of any skin manifestations usually seen in VZV reactivation [8]. Several months later, instead of showing signs of improvement as one would anticipate with viral encephalitis, the patient's condition deteriorated. This was evident, not only by his lumbar puncture confirming VZV, but also by a subsequent MRI showing more prominent inflammation in the leptomeningeal portions of the patient’s brain. 

The gold standard of diagnosing meningoencephalitis is a lumbar puncture. However, one is not always performed due to the risks involved with the procedure. This can include minimal adverse effects such as post-dural puncture headache and nerve irritation to more life-threatening infections and bleeding complications [9]. When a lumbar puncture is not performed during the acute onset of symptoms, because the mortality rate for encephalitis is a little over 50%, the diagnosis of meningoencephalitis is usually excluded from the differential diagnosis [10]. In our case, because a lumbar puncture was not performed, adequate treatment was delayed, and the patient was diagnosed with numerous other long-term conditions, such as illness anxiety disorder, which research showed to be seven times more prevalent in medical students than those not involved in the medical field [11]. 

Hence, we share this case to emphasize how VZV meningoencephalitis can manifest in various ways and the importance of keeping it on your differential diagnosis as a chronic condition, and not just in acute settings. There is very little, if any, information in the literature about patients suffering from chronic VZV meningoencephalitis, which reflects the need to do further research. To understand its prevalence in the population, and determine the risk-to-benefit analysis on performing a lumbar puncture to possibly diagnose a chronic VZV infection.

Patient perspective

As a second-year medical student and the subject of this case report, I, MZ (the first author), found that phrases like "look for horses instead of zebras" (a reminder to consider common diagnoses before rare ones) and "medical student syndrome" (the tendency for students to believe they have the diseases they’re studying) were emphasized and echoed throughout the whole experience. While it was frustrating to hear, I cannot deny the research showing that hypochondria affects medical students so much more than the general population. 

Regardless of how long I had to endure this illness, I am grateful to look back and ponder the interesting double-sided view I have gained from this experience. Still, I often find myself revisiting the lumbar puncture I was offered during my first emergency room visit over and over. The attending physician believed I was still battling a middle ear infection and presented the lumbar puncture as a kind of haphazard option; however, he ultimately recommended against it while painting a picture of the accompanying severe risks and side effects. Though unsure of the correct sequence of events due to my significantly altered mental state at the time, I still wonder if I should have said yes to the procedure, or if it should have even been offered at all, given that my physician ultimately recommended against it.

That being said, I now understand the struggle and frustration an undiagnosed patient can go through, but on the other hand, as a medical student, I appreciate why it took so long to be diagnosed based on the vagueness and unusual sequelae of symptoms I presented with. In terms of my headaches, dizziness, tinnitus, and brain fog, I cannot say they have disappeared entirely. Brain fog in of itself is neuroinflammation after all, and while medication may help, I understand that it will take time for this to fully go away. However, that being said, I am truthfully grateful to say that I am feeling a lot better and that I have the cognitive capacity to help write this paper, and leave this message to my peers and future clinicians.

## Conclusions

VZV syndrome can present with nonspecific neurologic symptoms and should be considered in the differential diagnosis for patients with indolent symptoms without any characteristic dermatomal skin rash or leukocytosis. From the present presentation, we can see that these symptoms can range from a GI presentation, to ocular, to auditory, and even to psychiatric. While further research should be performed to assess where this diagnosis should fall when considering the existing algorithm, we urge our peers and future physicians to begin considering VZV syndrome as a diagnosis for chronic symptoms, at the very least by exclusion of other pathologies. Therefore, we propose that a lumbar puncture should be considered as the benefits of addressing such a chronic condition far outweigh the risks and complications associated with the procedure.

## References

[1] Gershon AA, Breuer J, Cohen JI (2015). Varicella zoster virus infection. Nat Rev Dis Primers.

[2] Koskiniemi M, Piiparinen H, Rantalaiho T (2002). Acute central nervous system complications in varicella zoster virus infections. J Clin Virol.

[3] Ibraheem M, Marin M, Leung J (2013). Fatal wild-type varicella-zoster virus encephalitis without a rash in a vaccinated child. Pediatr Infect Dis J.

[4] Mirouse A, Sonneville R, Razazi K (2022). Neurologic outcome of VZV encephalitis one year after ICU admission: a multicenter cohort study. Ann Intensive Care.

[5] Lizzi J, Hill T, Jakubowski J (2019). Varicella zoster virus encephalitis. Clin Pract Cases Emerg Med.

[6] Le N, Razick DI, Dhaliwal A, Akhtar M, Daniel E (2023). A rare case of varicella-zoster virus encephalitis presenting with lost ability to play the piano in an immunocompetent pediatric patient. Cureus.

[7] Alvarez JC, Alvarez J, Tinoco J (2020). Varicella-zoster virus meningitis and encephalitis: an understated cause of central nervous system infections. Cureus.

[8] Takami K, Kenzaka T, Kumabe A (2022). Varicella-zoster virus-associated meningitis, encephalitis, and myelitis with sporadic skin blisters: A case report. World J Clin Cases.

[9] Evans RW (1998). Complications of lumbar puncture. Neurol Clin.

[10] Said S, Kang M (2024). Viral encephalitis. StatPearls.

[11] Sherif HA, Tawfeeq K, Mohamed Z (2023). “Medical student syndrome”: a real disease or just a myth?—a cross-sectional study at Menoufia University, Egypt.

